# Perceptual Grouping without Awareness: Superiority of Kanizsa Triangle in Breaking Interocular Suppression

**DOI:** 10.1371/journal.pone.0040106

**Published:** 2012-06-29

**Authors:** Lan Wang, Xuchu Weng, Sheng He

**Affiliations:** 1 Institute of Psychology, Chinese Academy of Sciences, Beijing, China; 2 Graduate University of Chinese Academy of Sciences, Beijing, China; 3 Center for Cognition and Brain Disorders, Hangzhou Normal University, Hangzhou, Zhejiang, China; 4 Department of Psychology, University of Minnesota, Minneapolis, Minnesota, United States of America; Queen Mary University of London, United Kingdom

## Abstract

Much information could be processed unconsciously. However, there is no direct evidence on whether perceptual grouping could occur without awareness. To answer this question, we investigated whether a Kanizsa triangle (an example of perceptual grouping) is processed differently from stimuli with the same local components but are ungrouped or weakly grouped. Specifically, using a suppression time paradigm we tested whether a Kanizsa triangle would emerge from interocular continuous flash suppression sooner than control stimuli. Results show a significant advantage of the Kanizsa triangle: the Kanizsa triangle emerged from suppression noise significantly faster than the control stimulus with the local Pacmen randomly rotated (*t*(9) = −2.78, *p* = 0.02); and also faster than the control stimulus with all Pacmen rotated 180° (*t*(11) = −3.20, *p*<0.01). Additional results demonstrated that the advantage of the grouped Kanizsa triangle could not be accounted for by the faster detection speed at the conscious level for the Kanizsa figures on a dynamic noise background. Our results indicate that certain properties supporting perceptual grouping could be processed in the absence of awareness.

## Introduction

A large part of the visual information processing is outside of awareness. What is the capacity of the unconscious visual information processing? Answers to this question are likely context dependent. Continuous flash suppression or interocular suppression provides one way to render a visually presented stimulus invisible [1], [2], and studies have shown that processing of some low level features such as orientation [3], color [1], and luminance [4] can survive interocular suppression. However, the extent of unconscious processing of higher-level information remains unclear. While some special aspect of high level information such as facial expression and manipulable objects can survive interocular suppression [5], [6], [7], [8], it seems they may be processed through special pathways (e.g., subcortical pathway) rather than the typical stages of object recognition [9], [10]. In contrast, other types of high-level information (for example, race, gender and high-level shape aspect of face) were not processed when rendered invisible by continuous flash suppression [11], [12]. Therefore, whether some basic operations involved in conventional object processing could occur during interocular suppression remains an open question. In the present work, we investigated whether an important process in object perception, namely perceptual grouping, could occur in the absence of awareness.

Perceptual grouping serves to bring together components likely belonging to a common cause, such as the same contour, surface or object [13]. It is closely related to the surface segmentation process as discontinuity often arises from surface occlusions. Some studies suggest that detection of texture discontinuity could occur pre-attentively [14], [15], but perceptual grouping based on proximity and similartiy cues do compete for attention [16]. However, a notable study by Moore and Egeth [17] showed that “before attention is allocated within a scene, visual information is parsed according to the Gestalt principles of organization”. In this particular case, grouping is based on luminance contrast, and according to the authors “the grouping patterns were quite salient” [17]. Thus certain forms of perceptual grouping may occur pre-attentively. These studies suggest the possibility that perceptual grouping may occur in the absence of awareness as well, although we should note that attention and awareness are two related but distinct processes [18], [19], [20]. It remains interesting to investigate whether perceptual grouping could occur in the absence of awareness.

We used a suppression time paradigm to directly investigate unconscious grouping. Similar to Continuous Flash Suppression (CFS) [1], [2], this suppression time measurement is also a variant of binocular rivalry. In CFS, by continuously flashing a series of different high-contrast and contour-rich random patterns to one eye, the information presented to the other eye can be suppressed for a relatively long time. In the suppression time paradigm, the contrast of the test image gradually ramps up so that it will break the flash suppression at some point in time. By initially rendering a stimulus invisible under interocular suppression and then measuring the time it takes for the stimulus to gain perceptual dominance, this suppression time paradigm provides an index on whether different types of visual information are differentially processed in the absence of awareness [6]. Commonly, the different time of suppression is compared to the potentially different time of detection when the stimulus is presented binocularly and blended into the noise with gradually increasing contrast. The purpose of this comparison is to check whether the different response time in the suppression condition could be accounted for by response bias or any other potential factors during conscious processing. This paradigm has been used to demonstrate, among other properties, that the visual system is sensitive to the face orientation (upright vs. inverted) in the absence of awareness, in that an upright face came out of suppression sooner than an inverted face, and there was no significant difference in detection time when an upright or inverted face is blended into the noise and viewed binocularly [6] (See [21] for a detailed discussion of this approach).

**Figure 1:**
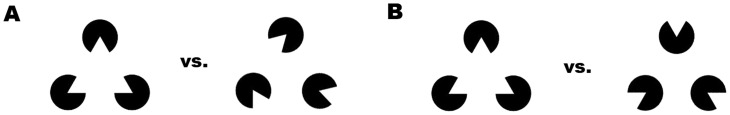
Illustration of the stimuli (A) Stimuli used in experiment 1: Kanizsa triangle and the control stimulus with the local Pacmen randomly rotated; and (B) Stimuli used in experiment 2: Kanizsa triangle and the symmetry control stimulus with each of the local Pacmen rotated 180°.

In this study, we used a Kanizsa figure [11] as a test example of grouping. The advantage of using a Kanizsa figure in this study is that its global grouping can be destroyed without changing the low-level properties of the image. This is critical in the suppression time paradigm since the depth of interocular suppression is sensitive to the low-level image features, such as luminance, color, size and so on [22]. As shown in figure 1, when the inducers (Pacmen) were oriented with the gaps forming the three corners of a triangle, observers could see an illusory white triangle (Kanizsa triangle) on top of three black discs. When the orientations of the inducers were altered (random rotation in Fig. 1A and systematic 180° rotation in Fig. 1B), the percept of the illusory triangle would disappear and the link between the three Pacmen would be much weaker, at the same time each individual local Pacman remain the same. The key point here is that the rotation of the local Pacmen changed the grouping between them without changing their local image properties. So we are able to probe the operation of perceptual grouping between local elements by contrasting the Kanizsa figure with the corresponding ungrouped stimuli.

We measured the time needed for a stimulus to break from suppression in two separate experiments. In the first experiment, we compared the response time of the Kanizsa triangle and the control stimulus with the Pacmen randomly rotated, both in an interocular suppression condition and in a binocular control condition. Results from this experiment will inform us on whether the Kanizsa figure and the random control were processed differently during suppression. Because the Kanizsa figure is symmetric while the randomly rotated control is not, we further investigated the contribution of symmetry in the second experiment by comparing the Kanizsa triangle with a control stimulus in which all Pacmen were rotated 180°.

Logically, if one stimulus is detected sooner than another in the suppression time experiment, it is possible that the difference is caused by differential sensitivity to the stimuli either before or after they emerge from suppression. To measure how much, if any, advantage a Kanizsa figure has over the control stimuli in terms of detection at the conscious stage, we also ran a binocular control experiment for each suppression experiment. In the control experiment, the same Kanizsa triangle and its control figure were blended into the dynamic noise pattern and presented binocularly. In order to make the reaction time in the binocular control condition and the experiment condition fall in the similar range for a fair comparison, the contrast of test images was ramped up much slower in the binocular control condition.

## Methods

### Ethics Statement

The experimental procedure was approved by the IRB of the University of Minnesota. All participants provided written, informed consent before taking part in the experiment.

### Participants

Ten observers (6 females) whose age ranged from 21 to 30 participated in experiment 1, and another group of twelve observers (10 female) whose age ranged from 18 to 24 participated in experiment 2. They had normal or corrected-to-normal visual acuity.

### Procedure

#### Experiment 1

Stimuli were presented on an Intel Coro2 Duo 3.16 GHz computer driving a 19-in CRT monitor at a resolution of 1024×768 pixels. Responses were gathered with a standard keyboard. The experiment was controlled using MatLab and the Psychophysics Toolbox [13], [14]. The images presented to the two eyes were displayed side-by-side on the monitor and fused using a mirror stereoscope mounted on a chin rest. A frame (11.3°×11.3°) that extended beyond the outer border of the stimulus and fixation point was presented to facilitate stable convergence of the two images. The viewing distance was approximately 60 cm. The luminance of background was 0.96 cd/mm^2^, and the luminance of the Pacman was 0.31 cd/mm^2^.

**Figure 2:**
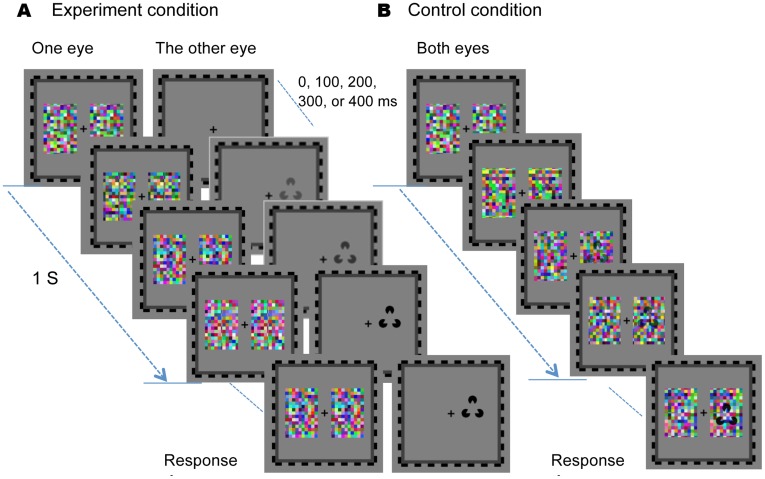
Schematic representation of the experimental paradigm In the experimental condition (A), a test figure was gradually introduced to one eye to compete with dynamic noise presented to the other eye. The test image was presented from 0, 100, 200, 300 or 400 ms after the trial began, with its contrast linearly ramped up from 0 to 100% within a period of 1 s, and then remained constant until the observer made a response to indicate on which side something other than noise appeared. In the control condition (B), a test image was presented directly on the noise background with its contrast increased gradually at a slower rate than in the experimental condition. Observers viewed the stimulus binocularly and responded to the appearance of the test image as soon as possible.

We had two blocks in experiment 1: one dichoptic presented suppression condition, and one binocular control condition. Figure 2 shows the general paradigm for the experimental procedures. In the experimental dichoptic presentation condition (Fig. 2A), a standard dynamic noise pattern was presented to one eye at full contrast throughout each trial, while the test figure was gradually introduced to the other eye at an uncertainty onset time (0, 100, 200, 300 or 400 ms from the beginning of the trial). The contrast of the test figure was ramped up gradually from 0 to 100% within a period of 1 s and then remained at full contrast until the observer made a button-press response to indicate on which side something emerged from noise. In the binocular control condition (Fig. 2B), a test image was presented directly on the noise background with its contrast increased gradually at a much slower rate (over a period of 10 s) than in the experimental condition. The reason for this slower ramping speed is to make the overall reaction time in the binocular control condition and the interocular suppression condition fall in the similar range for a fair comparison. In other words, if the detection time in the binocular control condition were much shorter than that in the interocular suppression condition, then there would be little room for potential detection advantage of one figure over another to manifest. The location of the test figure was random within the region corresponding to the location of the noise. A central cross (0.6°×0.6°) was always presented to each eye, serving as the fixation point.

The test images were Kanizsa triangle and a control stimulus, with the control stimulus generated by rotating each of the three local Pacmen randomly (Fig. 1A). Test image subtended (2.3°×1.9°) visual angles and was presented either to the left or to the right of fixation randomly. The horizontal distance between the center of the test image and fixation ranged from 1.9° to 2.9°, and the vertical center of the test image was anywhere between 2.9° above and 2.9° below fixation. At the very beginning of each trial, observers perceived the noise patch and were unaware which side contained the test image. They were asked to press the left or the right arrow key on a standard keyboard to indicate on which side of the fixation the test image appeared. They were told that they should respond to the appearance of any part of the test image as soon as possible and that they did not need to know the specific content of the image.

#### Experiment 2

The procedure was identical to Experiment 1, with the only exception that the random control stimulus was replaced by a symmetry figure produced by rotating all the ‘inducers’ in Kanizsa figure by180° (Fig. 1B). We also had a block of dichoptic presented suppression condition, and a block of binocular control condition in this experiment. In the second experiment we used a 17-in CRT monitor at a resolution of 1024×768 pixels, with visual angle and luminance of stimuli matching those in the first experiment.

In each experiment, the dichoptic suppression block and the control binocular block were run separately with the order counterbalanced across subjects.

## Results

**Figure 3:**
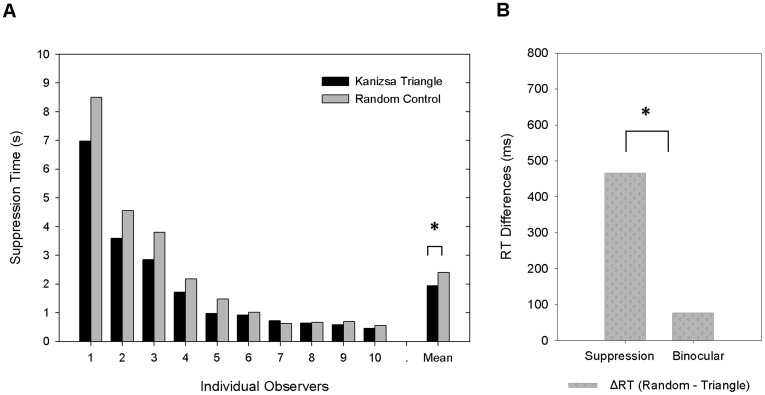
Results of Experiment 1, comparing the response time to the Kanizsa triangle and to the randomly rotated control stimulus. (A) Suppression times for the two types of images plotted for each of the 10 individual observers as well as their averages. The suppression time for the Kanizsa triangle is significantly shorter than that of the control stimulus, *p*<.05; (B) The advantage of Kanizsa triangle over the randomly rotated control stimulus, expressed as ΔRT, in the dichoptic suppression condition and in the binocular control condition. Advantage of Kanizsa triangle is significantly larger in the suppresion condion than that in the binocular control condition (466 ms vs. 77 ms, *t*(9) = 2.330, *p*<0.05).

We measured the time for a Kanizsa triangle and the corresponding ungrouped control stimuli to emerge from interocular noise suppression. In experiment 1, a significant superiority of Kanizsa triangle was found: A Kanizsa triangle took less time to emerge from the suppression noise than the control stimulus with the local Pacmen randomly rotated (466 ms shorter, 1938 ms vs. 2404 ms, *t*(9) = −2.78, *p* = 0.02) (Fig. 3A). This result suggests that the Kanizsa triangle was more potent than its ungrouped control stimulus against the suppression noise while they were suppressed from awareness.

We also ran a binocular control experiment and measured the potential detection advantage or response criterion difference for the Kanizsa figure over the control stimulus on noise background. Results from the binocular control experiment showed a slight but significant advantage for the Kanizsa figure (77 ms shorter, 1677 ms vs. 1754 ms, *t*(9) = −4.35, *p* = 0.002). However, the different suppression time in the experimental (interocular suppression) condition could not be explained simply by different detection times for the two types of stimuli, for the following reasons. First, the difference of RTs between detecting Kanizsa triangle and its control in the binocular viewing experiment was much smaller than the RT difference in the experimental dichoptic viewing condition (77 ms vs. 466 ms, 6 times larger in experiment condition, *t*(9) = −2.330, *p* = 0.045) (see Fig. 3B), and a joint analysis of the CFS and binocular condition in a two-way repeated measures ANOVA showed a significant interaction between experiment condition (dichoptic, binocular) and stimulus type (Kanizsa, random rotated control), *F* (1, 9) = 5.43, *p*<.05, which indicates there was additional benefit from grouping effect in the interocular suppression condition beyond that in the binocular conditions. Further, there is no significant correlation (*r* = 0.079, *p* = 0.829) between the RTs recorded in suppression experiment and in the control experiment across individuals, providing additional support that the advantage of the Kanizsa triangle in the suppression time condition is independent of its fast detection in the binocular control condition.

**Figure 4:**
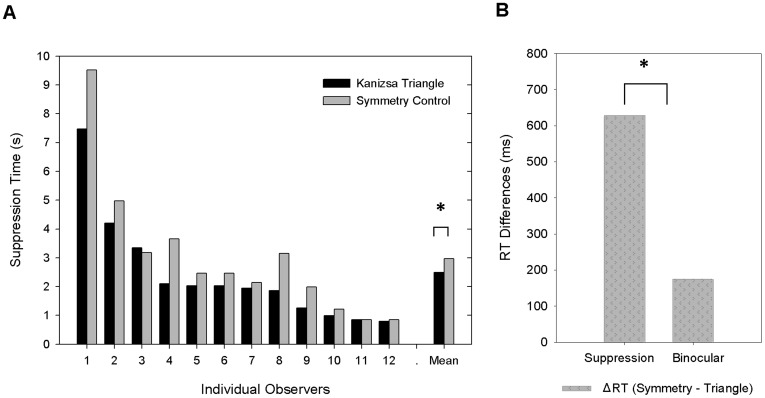
Results of Experiment 2, comparing the response time to the Kanizsa triangle and to the symmetry control stimulus. (A) Suppression times for the two types of images plotted for each of the 12 individual observers as well as their averages. The suppression time for the Kanizsa triangle is significantly shorter than that of the control stimulus, *p*<.05; (B) The advantage of Kanizsa triangle over the symmetry control stimulus, expressed as ΔRT, in the dichoptic suppression condition and in the binocular control condition. Advantage of Kanizsa triangle is significantly larger in the suppresion condion than that in the binocular control condition (629 ms vs. 175 ms, *t*(11) = 2.559, *p*<0.05 ).

In experiment 2, we also found a significant superiority of the Kanizsa triangle in the suppression condition: the Kanizsa triangle took less time to emerge from the suppression noise than the control stimulus that maintained symmetry (629.2 ms shorter, 2409 ms vs. 3038 ms, *t*(11) = −3.198, *p*<0.01) (Fig. 4A), and the Kanizsa figure also had a significant but smaller advantage over the control stimulus in the binocularly viewed control condition (174.5 ms shorter, 2132 ms vs. 2307 ms, *t*(11) = −4.917, *p*<0.01). A joint analysis of the CFS and binocular condition in a two-way repeated measures ANOVA again showed significant interaction between experimental condition (dichoptic, binocular) and stimulus type (Kanizsa triangle, symmetry control), *F* (1, 11) = 6.757, *p*<.05. This pattern of result suggests that the Kanizsa triangle was more potent than the symmetry control stimulus against the suppression noise while they were suppressed from awareness, and the different response time in the interocular suppression condition could not be accounted for simply by a small detection advantage for the Kanizsa figure than its control on a dynamic noise background. In this experiment, the response time advantages in the dichoptic and binocular are significantly correlated (*r* = 0.67, *p* = 0.02), which suggests that there might be a shared component in the dichoptic and binocular conditions that contributed to the fast response to the Kanizsa triangle. Still the benefit in detection is not sufficient to account for the suppression time advantage, as shown by the interaction and the larger magnitude of the advantage in suppression condition (629 ms vs. 175 ms, *t*(11) = 2.559, *p*<0.05) (Fig. 4B).

## Discussion

The present results demonstrated that a Kanizsa triangle emerged faster from interocular suppression than control figures consisting of the same local Pacmen but without strong link between the local elements. Further binocular control experiment showed that the advantage of the Kanizsa figure in competing against suppression noise could not be accounted for by the small detection advantage of the Kanizsa figure. A direct and straightforward characterization of these results is that it is faster for the Kanizsa figure to gain access to awareness, or the Kanizsa figure is a more potent stimulus in competing against the suppression noise [21]. These results then imply that the Kanizsa figure and the rotated control stimuli were processed differently, likely because that some form of grouping could occur during interocular suppression.

A recent study reported that observers could not discriminate the facing direction of illusory triangles when the inducers were rendered invisible through interocular suppression [23]. Such an observation may appear on the surface to be inconsistent with our current finding. However, the requirements for *explicitly* perceiving the illusory contour between the invisible inducing Pacmen can go much beyond that of perceptual grouping without awareness. In any case, the failure to explicitly perceive the illusory contour does not necessarily mean that perceptual grouping could not occur under interocular suppression. In the present study, we adopted a possibly more sensitive measure of unconscious processing and showed that the Kanizsa triangle and the ungrouped control figures were processed differently during interocular suppression.

What is the implication of our finding? In a simplistic way, this result suggests that the neural sites of perceptual grouping precede the cortical site of interocular suppression in the visual information processing hierarchy. Neural correlates of binocular rivalry have been found at multiple stages of visual processing, including the primary visual cortex [24], [25], [26], extrastriate visual cortex [24], [25], [27], as well as fusiform cortex [28], and even in the human lateral geniculate nucleus [29], [30]. A recent study by Watanabe et al. [19] dissociated selective attention from visual consciousness, and their conclusions support the idea that the cortical site of interocular suppression is beyond primary visual cortex. Thus it is well accepted that binocular suppression operates at multiple levels of the visual pathway [31], [32], [33], [34]. Our result is compatible with this view, suggesting that the process for perceptual grouping is at least not located after the sites of interocular suppression.

Results from experiment 2 show a significant advantage of the Kanizsa triangle over the symmetry control stimulus in breaking from suppression, which means that before the stimuli gained dominance and entered awareness, the visual system registered additional information about the Kanizsa triangle beyond its overall symmetrical configuration. The binocular control experiment also showed a detection advantage for the Kanizsa figure over the symmetry control stimulus, but the magnitude of the advantage in the binocular condition is not sufficient to account for the faster response time for the Kanizsa figure in the dichoptic condition.

While our results show that some aspects of perceptual grouping could occur under interocular suppression, they do not constitute as direct evidence for the neural representation of the subjective contours under suppression. It has been suggested that the neural events underlying rivalry suppression precede those underlying the synthesis of subjective contours. For example, rivalry suppression reduced the magnitude of the tilt aftereffect when the adapting and test patterns are subjective contours [35], and suppression is unaffected by a moving subjective contour whereas the formation of a subjective contour is impaired as indexed by the contour’s failure to enhance probe detection [36]. It is possible that under interocular suppression, some aspect of the perceptual grouping process occurred which made a difference in suppression time, but may not lead to a full representation of the illusory contour. Future studies with stimuli based on more traditional Gestalt grouping principles may provide more specific insights on what type of perceptual grouping could occur without awareness.

Closely related to perceptual grouping is the process of surface segmentation, especially in the case of Kanizsa figures, since normally the final perceptual outcome of a Kanizsa figure is the perception of a subjective surface partially occluding a number of local elements. Surface segmentation could lead to an integrated, partial object representation in the lateral occipital complex [37], [38], independent of the availability of attentional resources [39], and the presence of salient surface information have been shown to influence the efficiency of target detection [37], [40]. Although it is possible that the surface segmentation mechanism is engaged for the Kanizsa figure under interocular suppression, our results are only suggestive regarding this possibility and we cannot draw a firm conclusion regarding this possibility. Such a question will be better answered with neuroimaging measures in the future. It should also be noted that the current result is obtained with a particular type of perceptual grouping, one that is afforded by collinear boundaries supporting a subjective occlusion interpretation (i.e., a triangle partially occluding three discs). Whether perceptual grouping based on other properties (e.g., similarity, common fate, etc.) could occur in the absence of awareness remains an open question.

In conclusion, this study showed that a Kanizsa triangle could break from interocular noise suppression faster than control stimuli, even though they all consist of the same local Pacmen. The difference between the Kanizsa figure and the control stimuli are in the relationship between the Pacmen. Thus some form of perceptual grouping occurred for the Kanizsa figure during interocular suppression. This result argues against a strict view of sequential operation with the cortical site(s) for perceptual grouping located after the sites of interocular suppression. Instead, our finding suggests that the two processes involve overlapping processing stages, allowing part of the perceptual grouping process operating under interocular suppression.
